# Association between School Performance and Anemia in Adolescents in Mexico

**DOI:** 10.3390/ijerph17051466

**Published:** 2020-02-25

**Authors:** Alejandro Mosiño, Karen P. Villagómez-Estrada, Alberto Prieto-Patrón

**Affiliations:** 1 División de Ciencias Económico Administrativas, Universidad de Guanajuato, CP. 36250 Guanajuato, Mexico; krn_vies@hotmail.com; 2 Nestlé Research Center, Vers-chez-les-Blanc, PO Box 44, 1000 Lausanne 26, Switzerland; Alberto.PrietoPatron@rdls.nestle.com

**Keywords:** anemia, education, ENSANUT, logit models

## Abstract

In school age children and adolescents, anemia might cause lower cognitive function and attention span, which in turn could diminish human capital accumulation. As children born in low-income households are more likely to be anemic, this may prevent many individuals from overcoming the intergenerational poverty traps. In this paper, we used data from the Mexican National Health and Nutrition Survey 2012 and focused on a sample of adolescents between 12 and 19 years of age to study the relationship between attending school without delay—our proxy for school performance—and anemia. We found a statistically significant association between the two variables. If this relationship is causal, the economic burden linked to the loss of school years could well exceed the costs associated with programs aimed at reducing the prevalence of anemia in vulnerable populations. Our results provide additional support to the existing literature on anemia as a significant barrier to school achievement.

## 1. Introduction

Anemia is a condition in which the number of red blood cells is insufficient to meet the physiological requirements of humans [1]. There are a number of pathological causes related to this disease. In neonates and young infants, immune hemolytic disease, infection, and hereditary disorders are most common. In older children, acquired causes of anemia are more likely, particularly iron deficiency anemia. Anemia affects approximately 32.9% of the world population [2], with a higher concentration in low and middle-income countries.

The prevalence of anemia in Mexico was 23.3% in children between 1 and 4 years old, 10.1% in children between 5 and 11 years old, and 5.3% in adolescents between 12 and 19 years old [3]. Prevalence of anemia also differs by region. For instance, center and south Mexico are the most affected regions for adolescents between 12 and 19 years old. Iron deficiency has been identified as one of the most important nutritional causes of anemia in Mexican children [4,5], the observed prevalence of vitamin B12 and folate deficiencies are found to be relatively low [6], and a high percentage of the population younger than 12 years is found to suffer from iron and zinc deficiency and to have low serum concentrations of copper and magnesium [7].

In Mexico, results in reducing anemia have been positive [3]. This is mainly due to food fortification and supplementation strategies implemented by the government, such as Prospera and Liconsa [8]. Such results should provide an incentive to keep putting effort into planning and implementing measures to reduce its prevalence given the high impact on quality of life and future income that the disease brings [9,10].

Studies linking anemia to economic losses have targeted mainly infants and adults. A remarkable study by Lozoff et al. [11] documented an irreversible damage to cognitive development in anemic infants 6 to 23 months old associated with a loss of IQ at later ages. Basta et al. [12] estimated that iron supplementation to anemic sugar cane adult workers increased their productivity by 17%. Basta’s findings have been referred to as one of the main sources for modeling the production losses caused by anemia in adults through their lowered productivity. As for school children and adolescents, some studies have highlighted the relationship between anemia and learning capabilities. Webb and Oski [13,14] found that the administration of iron supplements to adolescent women with low iron levels considerably improved their performance in different verbal and memory learning tests. Murray-Kolb and Beard [15] further showed that improving ferritin (iron storage protein) levels in iron-deficient children substantially improved their academic performance. Walker et al. [16] found that girls affected by anemia are more likely to exhibit poor school performance, a low attention span, and other social problems, such as early sexual activity. In contrast, in Mexico, Vega-Franco et al. [17] failed to find a statistically significant relationship between hemoglobin and intelligence test results. In a recent analysis based on a randomized iron supplementation experiment in adolescents from a marginalized rural population in Peru, Chong et al. [18] concluded that iron deficiency represents a major barrier to human capital accumulation.

The aim of this study was to analyze and interpret the relationship between school performance and anemia using a sample of Mexican students between 12 and 19 years of age. To this end, we used the data collected by the National Health Survey 2012, ENSANUT 2012 [19] and implemented a series of logistic models. To the best of our knowledge, no other paper makes use of our database and approach. Indeed, while the majority of the studies present a clinical-experimental approach, we used econometric techniques to analyze the data from the National Survey. Furthermore, our database provides information on schooling status and anemia that allowed us to deliver further insights with respect to the existing literature.

The rest of the article is organized as follows. Following this introduction, we present in Section 2 the data that we used to perform our analysis and our methodology. In Section 3, we present the main results of our study. In Section 4, we describe additional experiments we performed to bolster our main results. In Section 5 we discuss our results. In Section 6 we conclude.

## 2. Data and Methodology

### 2.1. Hypothetical Model

**Figure 1:**
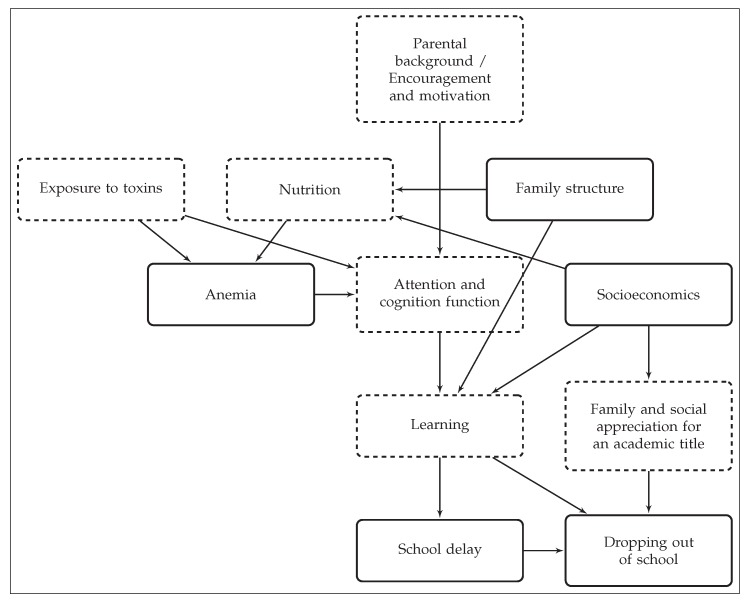
Causal diagram analysis of anemia and schooling outcomes. A simplifying model of how anemia, parental background, family structure, and socioeconomics could influence school delay and dropping out of school. Prepared by the authors.

In Figure 1, we illustrate how anemia, in combination with other factors, can influence schooling outcomes such as school delay and dropping out. From there, it is already evident that there is an empirical challenge to the estimation of a causal effect of anemia on schooling outcomes. Parental background, nutrition, family structure, environmental factors such as lead pollution, and socioeconomics most likely play a fundamental role in a given schooling system. Parental background includes genetic factors, wealth and family networks, and also encouragement and motivation from parents. Family structure and socioeconomic status of the household have an impact on nutrition. Nutrition, in turn, affects anemia status which can influence attention and cognition, therefore having an impact on the learning capabilities of the children. Proximity to toxic pollution can affect attention and cognition both directly [20,21] and indirectly through (hemolytic and aplastic) anemia [22]. Ultimately, learning ability likely affects both school delay and dropping out of school. The arrows show the direction of the effect. However, not all of the factors that we described are observed, at least in our data set. The dashed line rectangles show the unobserved factors that could potentially bias our results. For instance, cognitive functioning and adequate nutrition are expected to negatively affect school delay and dropping out of school and, at the same time, are positively correlated with socioeconomic conditions. This could imply that the effect of socioeconomics on school delay and dropping out of school could be underestimated. The solid line rectangles are factors that are at least partially observed and that could therefore be controlled for in our empirical model. We used Causal Diagrams Analysis to understand the association between anemia and schooling outcomes and the potential bias due to the unobserved factors described in the diagram. However, additional relationships in the model could exist, thus justifying the need for Structural Equation Modeling (SEM).

### 2.2. Study Population

The data we used in our analysis originates from ENSANUT 2012 [19]. This is a cross-sectional survey in which 50,528 households were interviewed, with a response rate of 87%. The survey contains extensive information regarding the households and their members, including the results of a hemoglobin (Hb) test using capillary blood. The ENSANUT 2012 [19] databases provide Hb concentration level measurement as well as a binary variable that takes the value of 1 if the individual has anemia and 0 if not. The diagnosis (presence or absence of anemia) was made using World Health Organization (WHO) criteria by adjusting Hb for altitude: the cut-off point is <120 g/L for men aged 12 to 14 and women over 12 years of age and <130 g/L for men over 15 years.

Our sample size of adolescents between 12 and 19 years of age is 10,835, after having excluded 200 women who declared they were pregnant at the time of the survey. The reason for the exclusion is that pregnancy simultaneously affects both school attendance and hemoglobin level, and therefore it could bias our results.

### 2.3. Dependent Variable

Our dependent variable is school attendance without delay as an indicator of educational performance. The ENSANUT 2012 [19] collected information if an individual was attending school at the time of the survey. In addition, using the exact age of the individual and the number of years of schooling, we deduced if the individual had any type of school delay. In order to do this, we determined the age of the individuals as of 31 December 2011. According to the Mexican education system, a child must be enrolled in primary school in September if she/he is six years old or will turn six before December 31. Generally, if a child does not repeat the year, the difference between his or her age and years of schooling completed by December 31 must always be six. There could be some exceptions, but it would be the minority of cases, as it would mostly include children born close to December 31. This is a binary variable that takes the value of one if the adolescent is attending school on the academic year he or she is supposed to and zero otherwise.

**Figure 2:**
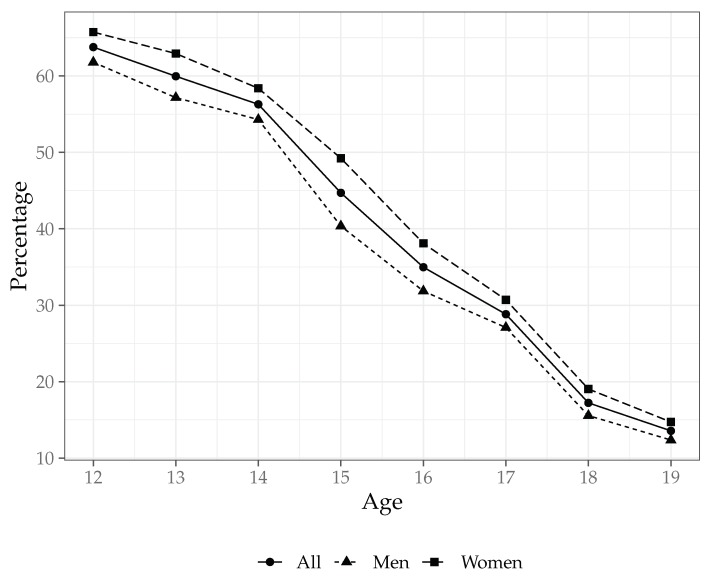
School attendance without delay by age and gender. Unweighted data. Prepared by authors.

Figure 2 shows how school attendance without delay varies with age and gender. Attendance without delay decreases from above 60% at 12 years old to about 15% at 19 years old. Adolescent girls attend school without delay in a larger proportion than boys. (In order to have further insight into the association between anemia and school attendance without delay, a complementary analysis is carried out in Appendix B. There, we ran a set of regression models using school attendance (with or without delay) as a dependent variable. Then, we use school attendance without delay but conditional on school attendance. Statistics of these variables are also shown in Table A1).

### 2.4. Explanatory Variables

Table A1 displays the descriptive statistics. The explanatory variables are divided into three groups: (1) anemia; (2) individual characteristics, which include sex, pregnancy rate (women who have ever been pregnant, excluding those who are currently pregnant), age, indigenous status, and work status (whether they are currently working or not); and (3) household and geographic characteristics, that include cohabitation with both parents, health insurance affiliation, socioeconomic level quintile, marginality index (poverty index at community level), and rural-urban classification and geographic region. Most vulnerable groups are expected to be at a disadvantage with respect to education indicators.

The sample is stratified by gender. It can be observed that women have a higher prevalence of anemia than men. This is a well-documented fact and is due to physiological changes in adolescent women during puberty. Another point to note is about 10% of the female adolescents reported having already had a pregnancy (excluding 200 women, 3.5%, who were pregnant at the time of the interview). This reflects the very high rate of early pregnancies in Mexico, which, together with several Latin American countries, has one of the highest rates in the world. Men show a higher rate of participation in the labor market than women, 16.7% versus 6.71%, as well as a higher cohabitation rate with both parents, 66.4% vs. 62.1%. The other variables are, in general, balanced across gender.

### 2.5. Statistical Analysis

In order to analyze the association between attending school without delay and our set of explanatory variables, we ran a series of logistic regressions. We firstly ran two separate regressions for women and men, then a third regression for the whole sample. This helped us to interpret the interaction between coefficients and gender.

As the ENSANUT 2012 [19] is a cross-sectional database, we cannot claim a causal relationship between our dependent variable and the different explanatory variables. (Causal relationships can be found in the case of a panel data analysis. However, the ENSANUT has anonymous observations, such as the panel cannot be built. A possible partial solution is to build a pseudo panel from the previous versions of the ENSANUT.) However, our analysis allowed us to obtain an initial picture of the association between our variable of interest and to develop a causality hypothesis based on the estimated magnitude. It is also important to stress that hemoglobin status is measured on the day of the interview whereas school delay may have occurred before. Therefore, the study relies on the assumption of persistent nutritional status. (Since the focus of our analysis is on anemia, our model can be less effective regarding the independent variables’ importance as predictors for school attendance. This is because the conceptual model was drawn for anemia and confounders were chosen accordingly.)

Our results are presented in the next section.

## 3. Results

Table A2 shows the estimates of the marginal coefficients from the three logistic regressions of school attendance without academic delay. We have chosen to give a global interpretation for the three groups of variables. Additionally, for a more straightforward intuition, we present in Table A5 the estimates in terms of odds ratios.

### 3.1. Anemia

Anemia is significantly associated with a lower probability of attending school without delay for men and women combined, with a coefficient of −0.326. In gender specific regressions, we found that the magnitude of the association is higher for men than for women, and it is statistically significant in both cases (−0.355 vs. −0.252). However, the difference of the magnitude between men and women was not statistically significant when we tested the interaction effect of anemia and gender in the pooled regression.

### 3.2. Individual Characteristics

Women were more likely than men to attend school without delay (0.269). Women who had a pregnancy had much lower probability to attend school without delay (−2489) than women who had not been pregnant. By looking at the magnitude of its coefficient, we observed that that the pregnancy variable is one of the most strongly correlated with almost all the education indicators. This suggests that reproductive control for young women is particularly relevant in terms of public policy.

We did not find a significant relationship between declaring oneself indigenous and attending school without delay for men and women taken together. However, in the women only regression, identifying as indigenous is significantly associated with a lower probability of attending school without delay (−0.155).

As expected, age is one of the factors that is most strongly negatively associated with school attendance without delay. In general, the probability of attending school without delay decreases with age, and the effect is stronger in men than in women.

Participation in the labor market is strongly associated with a lower probability of attending school without delay (−2043). This may reflect that having a job makes it more difficult for young people to continue studying but also that dropping out of school makes young people search for a job.

### 3.3. Home and Geographic Features

Teens who cohabit with both parents are significantly more likely to attend school without delay (0.173). Similarly, adolescents in more privileged households (higher socioeconomic levels) are more likely to attend school without delay.

Despite controlling for the economic variables, we find a strong geographic disparity in academic performance. The South is the region of the country where adolescents are least likely (−1.244 vs. North) to attend school without delay. There is also some disparity among adolescents from urban and metropolitan areas compared to adolescents from rural areas. Our results indicate that adolescents from urban and metropolitan areas are least likely to attend school without delay (−0.11 vs. rural), this effect is statistically significant for the whole sample and for men.

Finally, the probability of attending school without delay is lower for marginalized adolescents (−0.184).

## 4. Robustness Check

We tested the robustness of our results by running different models. By using as explanatory variable Hb concentration index rather than the binary variable anemia, we found similar results. Indeed, we can expect an individual with Hb concentrations above the cutoff value for anemia to perform better in school than individuals with lower concentrations. We also defined a series of binary variables for different levels of Hb concentration, from 0 to 11, from 11 to 12, from 12 to 13, from 13 to 14, from 14 to 15, and from 15 to infinity g/dL. By doing so, we could analyze the effect of different levels of Hb concentration and determine the point at which it affects school attendance without delay. Finally, while keeping the Hb concentration as explanatory variable, we restricted the sample to individuals with concentrations between 11 g/dL and 15 g/dL. This allowed us to study individuals that were more comparable. Table A6 reports the results of these three models.

The results in Table A6 confirm that the sign of the variable Hb is opposite to that obtained from the regressions in the previous section. In general, for an individual with higher concentrations of Hb, the probability of attending school without delay increases.

When we considered the series of binary variables for the different levels of Hb concentration, the highest probability to attend school without delay for women occurred in the levels between 12 g/dL and 15 g/dL. This is particularly interesting because 12 g/dL is precisely the cut-off point between having anemia or not. When we considered men and women together, we observed that their performance improves significantly above 12 g/dL and peak performance is reached at the levels between 15 g/dL and 16 g/dL. There is no evidence that different levels of Hb concentration affect men’s school performance.

When we restricted the sample to youth with Hb levels between 11 g/dL and 15 g/dL, we found no evidence of a significant relationship with any of the education indicators, except for the men sample. However, the signs of the full sample are generally maintained.

Although not shown in Table A6, signs, magnitudes and significance levels of the rest of the variables included in the models remained essentially constant in all exercises we performed.

Finally, signs and significance remained the same also when we created a variable for the distance of Hb concentration levels from the cutoff value for anemia and included it in the model together with the binary variable anemia. Results are not reported here.

## 5. Discussion

Our results are aligned with previous studies (See, for instance, Gaviria and Hoyos [23]) that showed a negative association between anemia and educational performance in adolescence. Additionally, we found that age, socioeconomic status, and teen pregnancy were strongly associated with attending school without delay.

We identified that the most vulnerable groups—indigenous women, highly marginalized people, and those in the lowest income quintiles—were associated with poorer educational indicators. Our analysis also shows that family structure and access to health services were significantly correlated with school performance. The participation in the labor market is significantly associated with a lower probability of attending school without delay. This intuitive result is in line with the socioeconomic reality of Mexico: many students need to work to support their families, and this causes them to lag behind in school and eventually drop out. We observed a high rate of early pregnancy in our Mexican sample and strong negative association between early pregnancy and attending school without delay. This effect is a combination of a significant increase in the probability both of dropping out of school and of grade repetition. Supporting adolescent women in decreasing the risk of early pregnancy, reducing the barriers to contraceptive access, could result in large economic benefits because of the implications for human capital accumulation [24].

Our model formalizes certain results that have been traditionally obtained, at least in Mexico, through observation and intuition. It therefore constitutes a justification for government programs aimed at reducing some of the main social problems afflicting the country, particularly malnutrition.

According to the International Program for the Evaluation of Students (PISA) 2012 [25], Mexico lags behind the countries participating in the PISA evaluation both in terms of average years of schooling and of quality of education. Most of the reasons behind poor performance in the PISA evaluation could be attributed to the educational system rather than to the nutritional status of the Mexican students. However, a recent meta-analysis of randomized trials aimed at improving learning in developing countries’ schools [26] showed how nutritional interventions primarily aimed at improving micronutrient status have a favorable effect. Specific recommendations of the Pan American Health Organization (PAHO) and the World Health Organization (WHO) [27] in this regard include iron fortification, supplementation, nutritional promotion, communication, and education, as well as nutritional epidemiological surveillance. Other strategies may include subsidies for the production and consumption of fortified foods, programs for direct food distribution to households, school breakfasts, distribution of vitamin A and micronutrient supplementation in dietary doses, and fortification of foods with micronutrients targeting specific populations. Recent programs implemented by the Mexican government include the OPORTUNIDADES Program, the Food Support Program (PAL), the Social Milk Abatement Program by Liconsa SA de CV, and the Rural Food Support Program led by Diconsa SA de CV.

Our study also reveals the importance of ensuring total (effective) coverage in terms of health services, of implementing public policies aimed at promoting family union, of providing information to adolescent men and women to reduce the number of unwanted pregnancies, and of granting support to adolescent men and women in order for them not to leave school to search for a job. The decline—if not the eradication—of education problems also depends on the policies applied by the states that make up the different Mexican regions, particularly those designed to reduce the disadvantages faced by women and to ensure full access to health and food services in rural and the most marginalized areas.

There are some limitations to this study. First, given the nature of the data, we are able to hypothesize why the association between educational performance and anemia is actually observed, but we do not have the data framework to claim a causal path. Second, hemoglobin status is measured on the day of the interview whereas school delay may have occurred before. Therefore, the study relies on the assumption of persistent nutritional status (an individual who was diagnosed with anemia in a given day is more likely to have had previous episodes of anemia in the past). Third, reverse causality could be an issue if being delayed at school or attending school affected the nutritional status of adolescents. Cohort studies could help in reaching better clarity in this kind of analysis, such as the very recent study of Wedu et al. [28]. Finally, there are a list of variables that are not available in our database. These include environmental factors, such as exposure to toxins, that can affect attention and cognition both directly and indirectly.

## 6. Conclusions

In this article, we have analyzed the relationship between attending school without delay and anemia using the Mexican student population between 12 and 19 years of age. We have run a set of regressions of school attendance without delay on anemia and confounding factors. In contrast with the existing literature—in which the relationship between student performance and anemia has been studied from a clinical-experimental perspective—we used an econometric approach. In particular, as we dealt with categorical variables, we implemented a series of logistic models. Our database also allowed us to incorporate into the analysis several socioeconomic factors such as age, gender, geographic location, rural-urban classification, indigenous status, among others.

Our main result is that there is a significant association between schooling outcomes and anemia in Mexican adolescents. Most of the studies analyzing the potential consequences of anemia in adolescents use cognitive tests’ measurements. There exists a substantial literature on returns on education linking years of education and wages but not yet on cognitive measurements. Thus, having parameters associating anemia and schooling outcomes facilitates building health-economics models to estimate the burden of anemia and to evaluate the cost effectiveness of programs aimed at reducing anemia in this particular age group. Our paper, in spite of its limitations, brings additional evidence to help in the assessment of fortification and supplementation programs in developing countries.

## Abbreviations

The following abbreviations are used in this manuscript:

ENSANUT 2012	Mexican National Health and Nutrition Survey 2012
Hb	Hemoglobin (concentration)
SEM	Structural Equation Modeling
PAHO	Pan American Health Organization
WHO	World Health Organization
PAL	Mexican Food Support Program

## Appendix A. Additional Dependent Variables

As mentioned in the main text of this paper, we can have further insight on the association between anemia and school attendance without delay by running a set of regression models. First we used school attendance without delay but conditional on school attendance as a dependent variable, next we used school attendance (with or without delay). In Table A3 we show the results of the former, and in Table A4 we show results of the latter. The estimates in terms of odds ratios are shown in Table A5.

Some of the main conclusions we get from this analysis can be summarized as follows:

- Anemia is significantly associated with a lower probability of attending school without delay for men and women as a whole and the magnitude of the association is higher for men than for women. When we divide the effect of attending school without delay conditional on school attendance (Table A3) and on whether or not the student attends school (Table A4), we observe that anemia is significantly associated with a lower probability of being at school without delay (−0.313) for the sample as a whole. This probability is lower for men (−0.464) than for women (−0.233) and is statistically significant in both cases. In contrast, we did not find a significant association between anemia and school attendance in general. In the sample of men, however, anemia is significantly positively associated with attendance rate. This may appear to be a counterintuitive result; nevertheless, it could simply reflect the fact that men receive more support from their environment than women when it comes to obtaining a particular academic degree: if a boy repeats a grade because of anemia, he will tend to compensate for it with a greater number of years in school (higher attendance). This result therefore is not in contrast with our previous findings and can be a confirmation of the fact that men with anemia tend to be more delayed than women. Women, instead, are more likely to drop out. Another point to consider in interpreting this coefficient is that anemia is associated with lower productivity in manual labor [12]. Lower productivity due to anemia could potentially reduce the likelihood of young male employment and, therefore, the opportunity cost of attending school.
- Women who had a pregnancy had much lower probability of attending school without delay than women who had not been pregnant. According to Table A3 and Table A4 this effect can result from a combination of a lower probability of school attendance (−2.687) and, if they attend school, a lower probability of not being delayed (−0.748).
- Age is one of the factors that are most strongly positively associated with dropping out of school. Between the ages of 16 and 19, the effect is stronger in women than in men. However, the results in Table A3 suggest that, conditional on school attendance, males have a lower probability of attending school without delay than females. In general, the probability of attending school without delay decreases with age, and the effect is stronger in men than in women.
- Teens who cohabit with both parents are significantly more likely to attend school without delay. They are also more likely to keep attending school (0.273) and, if they already attend school, they have a higher probability of not falling behind (0.125).
- The South is the region of the country where adolescents are least likely to attend school without delay. The disadvantage of the South is confirmed by the magnitude of the coefficient in Table A3. The probability of school attendance without delay conditional on attendance is much lower in the South than in the North (−1.515).
- Adolescents classified as marginalized have a higher probability of dropping out (−0.330) and, if they are attending school, a lower probability of attending without delay (−0.117).
- When we considered the full range of Hb concentrations in men, not only the likelihood of attending school without delay increases with the Hb levels but also the likelihood of attending school without delay conditional on school attendance (0.084). This result suggests that anemic men may receive more support than women, and thanks to that they tend to remain in school a greater number of years.

## Appendix B.

**Table A1:** Descriptive statistics of the sample (mean value of the variable).

	(1)	(2)	(3)
	Men and Women	Men	Women
VARIABLES	N=10,835	N=5719	N=5782
**Dependent variables**			
Attendance without delay %	39.3	37.2	41.5
Attendance without delay (conditional) %	66.7	64.3	69.1
Attendance in school %	72.6	72.9	72.4
**Explanatory variables**			
**Nutritional status**			
Anemia %	6.2	4.02	8.35
**Individual characteristics**			
Sex (% of women)	50.3	0	100
Pregnancy %	4.67	0	9.29
Indigenous %	22.2	22.4	22.1
Age (in years)	15.27	15.23	15.3
Work %	11.7	16.7	6.71
**Home and geographic features**			
Cohabitation with both parents %	64.2	66.4	62.1
Health insurance %	77.4	76.8	77.9
Quintile of socioeconomic level	2.989	3.010	2.968
Marginality index	40.4	39.7	41
Urban-Rural			
*Rural %*	35.5	34.7	36.4
*Metropolitan %*	20.8	20.2	21.3
*Urban %*	43.7	45.2	42.3
Region			
*North %*	25.7	26.0	25.4
*Center %*	36.2	36.0	36.4
*Mexico City %*	5.15	5.18	5.12
*South %*	33	32.8	33.1

**Table A2:** Estimation of the marginal coefficient of the logistic regression of attendance without delay.

	Dependent Variable		
	Attendance without Delay		
	Men and Women (1)	Men (2)	Women (3)
**Nutritional status**			
Anemia	−0.326 ${}^{***}$	−0.355 ${}^{**}$	−0.252 ${}^{**}$
Anemia & Woman	0.065		
**Individual characteristics**			
Sex (woman)	0.269 ${}^{***}$		
Pregnancy	−2.489 ${}^{***}$		−2.512 ${}^{***}$
Indigenous	−0.067	0.016	−0.155 ${}^{*}$
Age (12 years)			
*13 years*	−0.260 ${}^{***}$	−0.253 ${}^{**}$	−0.267 ${}^{**}$
*14 years*	−0.305 ${}^{***}$	−0.385 ${}^{***}$	−0.221 ${}^{**}$
*15 years*	−0.675 ${}^{***}$	−0.826 ${}^{***}$	−0.527 ${}^{***}$
*16 years*	−1.167 ${}^{***}$	−1.307 ${}^{***}$	−1.028 ${}^{***}$
*17 years*	−1.081 ${}^{***}$	−1.208 ${}^{***}$	−0.956 ${}^{***}$
*18 years*	−2.021 ${}^{***}$	−2.031 ${}^{***}$	−2.006 ${}^{***}$
*19 years*	−2.276 ${}^{***}$	−2.258 ${}^{***}$	−2.277 ${}^{***}$
Work	−2.043 ${}^{***}$	−2.108 ${}^{***}$	−1.870 ${}^{***}$
**Home and geographic features**			
Cohabitation with both parents	0.173 ${}^{***}$	0.148 ${}^{**}$	0.192 ${}^{***}$
Health insurance	0.504 ${}^{***}$	0.645 ${}^{***}$	0.367 ${}^{***}$
Socioeconomic level (NSE q1)			
*NSE q2*	0.340 ${}^{***}$	0.370 ${}^{***}$	0.315 ${}^{***}$
*NSE q3*	0.609 ${}^{***}$	0.612 ${}^{***}$	0.608 ${}^{***}$
*NSE q4*	0.816 ${}^{***}$	0.866 ${}^{***}$	0.767 ${}^{***}$
*NSE q5*	1.338 ${}^{***}$	1.363 ${}^{***}$	1.327 ${}^{***}$
Marginality Index	−0.184 ${}^{***}$	−0.196 ${}^{**}$	−0.180 ${}^{**}$
Urban-Rural (Rural)			
*Metropolitan*	−0.110 ${}^{*}$	−0.163 ${}^{*}$	−0.073
*Urban*	−0.112 ${}^{*}$	−0.191 ${}^{**}$	−0.045
Region (North)			
*Center*	−0.578 ${}^{***}$	−0.535 ${}^{***}$	−0.630 ${}^{***}$
*Mexico City*	−0.019	−0.025	−0.020
*South*	−1.244 ${}^{***}$	−1.234 ${}^{***}$	−1.259 ${}^{***}$
Constant	0.052	0.017	0.369 ${}^{**}$
Observations	11,501	5719	5782
Log-likelihood	−5984.126	−2952.766	−3019.904
Akaike. Inf. Crit.	12,020.250	5951.532	6087.807
McFadden’s Pseudo $R^{2}$	0.7266	0.7299	0.7233

${}^{*}$*p*< 0.1; ${}^{**}$ *p*< 0.05; ${}^{***}$ *p*< 0.01.

**Table A3:** Estimation of the marginal coefficient of the logistic regression of attendance without delay conditional on school attendance.

	Dependent Variable		
	Attendance without Delay Conditional to Attendance		
	Men and Women (1)	Men (2)	Women (3)
**Nutritional status**			
Anemia	−0.443 ${}^{***}$	−0.464 ${}^{***}$	−0.233 ${}^{*}$
Anemia & Woman	0.202		
**Individual characteristics**			
Sex (women)	0.291 ${}^{***}$		
Pregnancy	−0.747 ${}^{**}$		−0.805 ${}^{**}$
Indigenous	−0.134 ${}^{**}$	−0.094	−0.180 ${}^{**}$
Age (12 years)			
*13 years*	−0.212 ${}^{***}$	−0.214 ${}^{*}$	−0.210 ${}^{*}$
*14 years*	−0.181 ${}^{**}$	−0.239 ${}^{**}$	−0.118
*15 years*	−0.363 ${}^{***}$	−0.529 ${}^{***}$	−0.199 ${}^{*}$
*16 years*	−0.789 ${}^{***}$	−1.000 ${}^{***}$	−0.587 ${}^{***}$
*17 years*	−0.607 ${}^{***}$	−0.789 ${}^{***}$	−0.417 ${}^{***}$
*18 years*	−1.438 ${}^{***}$	−1.524 ${}^{***}$	−1.346 ${}^{***}$
*19 years*	−1.713 ${}^{***}$	−1.696 ${}^{***}$	−1.704 ${}^{***}$
Work	−0.598 ${}^{***}$	−0.543 ${}^{***}$	−0.643 ${}^{**}$
**Home and geographic characteristics**			
Cohabitation with both parents	0.125 ${}^{**}$	0.133 ${}^{*}$	0.108
Health insurance	0.313 ${}^{***}$	0.438 ${}^{***}$	0.189 ${}^{**}$
Socioeconomic level (q1) (NSE q1)			
*NSE q2*	0.311 ${}^{***}$	0.359 ${}^{***}$	0.269 ${}^{**}$
*NSE q3*	0.544 ${}^{***}$	0.579 ${}^{***}$	0.512 ${}^{***}$
*NSE q4*	0.662 ${}^{***}$	0.782 ${}^{***}$	0.543 ${}^{***}$
*NSE q5*	1.032 ${}^{***}$	1.095 ${}^{***}$	0.987 ${}^{***}$
Marginality Index	−0.117 ${}^{**}$	−0.124	−0.129
Urban-Rural (Rural)			
*Metropolitan*	−0.218 ${}^{***}$	−0.281 ${}^{***}$	−0.172 ${}^{*}$
*Urban*	−0.106	−0.192 ${}^{*}$	−0.036
Region (North)			
*Center*	−0.720 ${}^{***}$	−0.658 ${}^{***}$	−0.796 ${}^{***}$
*Mexico City*	−0.208 ${}^{*}$	−0.216	−0.206
*South*	−1.517 ${}^{***}$	−1.487 ${}^{***}$	−1.555 ${}^{***}$
Constant	0.593 ${}^{***}$	0.521 ${}^{***}$	0.979 ${}^{***}$
Observations	8354	4167	4187
Log-likelihood	−5028.218	−2519.818	−2497.478
Akaike. Inf. Crit.	10,108.440	5085.636	5042.956
McFadden’s Pseudo $R^{2}$	0.6495	0.6572	0.6409

${}^{*}$*p*< 0.1; ${}^{**}$ *p*< 0.05; ${}^{***}$ *p*< 0.01.

**Table A4:** Estimation of the marginal coefficient of logistic regression school attendance.

	Dependent Variable		
	School Attendance		
	Men and Women (1)	Men (2)	Women (3)
**Nutritional status**			
Anemia	0.502 ${}^{**}$	0.462 ${}^{**}$	−0.180
Anemia & Woman	−0.714 ${}^{***}$		
**Individual characteristics**			
Sex (woman)	−0.010		
Pregnancy	−2.676 ${}^{***}$		−2.604 ${}^{***}$
Indigenous	0.141 ${}^{**}$	0.367 ${}^{***}$	−0.055
Age (12 years)			
*13 years*	−0.602 ${}^{***}$	−0.562 ${}^{**}$	−0.635 ${}^{***}$
*14 years*	−1.002 ${}^{***}$	−1.130 ${}^{***}$	−0.833 ${}^{***}$
*15 years*	−1.744 ${}^{***}$	−1.773 ${}^{***}$	−1.697 ${}^{***}$
*16 years*	−2.188 ${}^{***}$	−2.078 ${}^{***}$	−2.272 ${}^{***}$
*17 years*	−2.374 ${}^{***}$	−2.343 ${}^{***}$	−2.403 ${}^{***}$
*18 years*	−2.954 ${}^{***}$	−2.839 ${}^{***}$	−3.064 ${}^{***}$
*19 years*	−3.080 ${}^{***}$	−3.058 ${}^{***}$	−3.094 ${}^{***}$
Work	−2.684 ${}^{***}$	−2.688 ${}^{***}$	−2.277 ${}^{***}$
**Home and geographic features**			
Cohabitation with both parents	0.275 ${}^{***}$	0.160 ${}^{*}$	0.377 ${}^{***}$
Health insurance	0.720 ${}^{***}$	0.822 ${}^{***}$	0.639 ${}^{***}$
Socioeconomic level (NSE q1)			
*NSE q2*	0.225 ${}^{***}$	0.136	0.291 ${}^{**}$
*NSE q3*	0.517 ${}^{***}$	0.355 ${}^{***}$	0.667 ${}^{***}$
*NSE q4*	0.877 ${}^{***}$	0.628 ${}^{***}$	1.126 ${}^{***}$
*NSE q5*	1.621 ${}^{***}$	1.516 ${}^{***}$	1.714 ${}^{***}$
Marginality Index	−0.333 ${}^{***}$	−0.379 ${}^{***}$	−0.287 ${}^{***}$
Urban-Rural (Rural)			
*Metropolitan*	0.305 ${}^{***}$	0.318 ${}^{***}$	0.281 ${}^{**}$
*Urban*	−0.014	−0.079	0.037
North Region			
*Center*	0.022	0.050	−0.032
*Mexico City*	0.477 ${}^{***}$	0.529 ${}^{**}$	0.391 ${}^{*}$
*South*	0.320 ${}^{***}$	0.425 ${}^{***}$	0.213 ${}^{*}$
Constant	2.047 ${}^{***}$	2.027 ${}^{***}$	1.969 ${}^{***}$
Observations	11,501	5719	5782
Log-likelihood	−4191.033	−2038.017	−2126.861
Akaike. Inf. Crit.	8434.067	4122.035	4301.722
McFadden’s Pseudo $R^{2}$	0.8002	0.8096	0.7929

${}^{*}$*p*< 0.1; ${}^{**}$ *p*< 0.05; ${}^{***}$ *p*< 0.01.

**Table A5:** Estimation of the odds ratio, men and women.

	Dependent Variable		
	Attendance ...		
	W/O Delay (1)	W/O (Conditional) (2)	Global (3)
**Nutritional status**			
Anemia	0.722 ${}^{***}$	0.642 ${}^{***}$	1.652 ${}^{***}$
Anemia & Woman	1.067 ${}^{***}$	1.224 ${}^{***}$	0.490 ${}^{***}$
**Individual characteristics**			
Sex (woman)	1.309 ${}^{***}$	1.338 ${}^{***}$	0.990 ${}^{***}$
Pregnancy	0.083	0.474	0.069
Indigenous	0.935 ${}^{***}$	0.875 ${}^{***}$	1.151 ${}^{***}$
Age (12 years)			
*13 years*	0.771 ${}^{***}$	0.809 ${}^{***}$	0.548 ${}^{***}$
*14 years*	0.737 ${}^{***}$	0.834 ${}^{***}$	0.367 ${}^{**}$
*15 years*	0.509 ${}^{***}$	0.696 ${}^{***}$	0.175
*16 years*	0.311 ${}^{***}$	0.454 ${}^{***}$	0.112
*17 years*	0.339 ${}^{***}$	0.545 ${}^{***}$	0.093
*18 years*	0.133	0.237 ${}^{**}$	0.052
*19 years*	0.103	0.180	0.046
Work	0.130	0.550 ${}^{***}$	0.068
**Home and geographic features**			
Cohabitation with both parents	1.189 ${}^{***}$	1.133 ${}^{***}$	1.317 ${}^{***}$
Health insurance	1.655 ${}^{***}$	1.367 ${}^{***}$	2.055 ${}^{***}$
Socioeconomic level (NSE q1)			
*NSE q2*	1.405 ${}^{***}$	1.364 ${}^{***}$	1.253 ${}^{***}$
*NSE q3*	1.839 ${}^{***}$	1.724 ${}^{***}$	1.678 ${}^{***}$
*NSE q4*	2.262 ${}^{***}$	1.938 ${}^{***}$	2.405 ${}^{***}$
*NSE q5*	3.811 ${}^{***}$	2.806 ${}^{***}$	5.059 ${}^{***}$
Marginality Index	0.832 ${}^{***}$	0.889 ${}^{***}$	0.717 ${}^{***}$
Urban-Rural (Rural)			
*Metropolitan*	0.896 ${}^{***}$	0.804 ${}^{***}$	1.357 ${}^{***}$
*Urban*	0.894 ${}^{***}$	0.899 ${}^{***}$	0.986 ${}^{***}$
North Region			
*Center*	0.561 ${}^{***}$	0.487 ${}^{***}$	1.022 ${}^{***}$
*Mexico City*	0.981 ${}^{***}$	0.812 ${}^{***}$	1.611 ${}^{***}$
*South*	0.288 ${}^{***}$	0.219 ${}^{***}$	1.377 ${}^{***}$
Constant	1.053 ${}^{***}$	1.809 ${}^{***}$	7.589 ${}^{***}$
Observations	11,501	8354	11,501
Log-likelihood	−5984.126	−5028.218	−4191.033
Akaike. Inf. Crit.	12,020.250	10,108.440	8434.067
McFadden’s Pseudo $R^{2}$	0.7266	0.6495	0.8002

${}^{*}$*p*<0.1; ${}^{**}$*p*<0.05; ${}^{***}$*p*<0.01.

**Table A6:** Estimation of several alternative models.

	Men and Women (1)	Men (2)	Women (3)
**Attendance without delay**			
Hb [0,∞)	0.028 ${}^{*}$	0.059 ${}^{***}$	0.016
Hb categorical (reference [0,11])			
Hb (11,12]	0.618 ${}^{**}$	0.130	0.805 ${}^{***}$
Hb (12,13]	0.733 ${}^{***}$	0.478	0.830 ${}^{***}$
Hb (13,14]	0.756 ${}^{***}$	0.546	0.845 ${}^{***}$
Hb (14,15]	0.786 ${}^{***}$	0.663	0.836 ${}^{***}$
Hb (15,16]	0.795 ${}^{***}$	0.716 ${}^{*}$	0.815 ${}^{***}$
Hb (16,∞)	0.752 ${}^{***}$	0.705	0.662 ${}^{**}$
Hb [11,15]	0.039	0.102 ${}^{*}$	0.018
**Attendance without delay (conditioned)**			
Hb [0,∞)	0.036 ${}^{**}$	0.084 ${}^{***}$	0.005
Hb categorical (reference [0,11])			
Hb (11,12]	0.632 ${}^{**}$	0.012	0.917 ${}^{***}$
Hb (12,13]	0.748 ${}^{***}$	0.360	0.922 ${}^{***}$
Hb (13,14]	0.755 ${}^{***}$	0.502	0.880 ${}^{***}$
Hb (14,15]	0.781 ${}^{***}$	0.574	0.892 ${}^{***}$
Hb (15,16]	0.832 ${}^{***}$	0.724	0.863 ${}^{***}$
Hb (16,∞)	0.810 ${}^{***}$	0.738	0.674 ${}^{**}$
Hb [11,15]	0.038	0.123 ${}^{**}$	−0.002
**Global attendance**			
Hb [0,∞)	−0.010	−0.051 ${}^{*}$	0.038
Hb categorical (reference [0,11])			
Hb (11,12]	0.182	1.073	0.001
Hb (12,13]	0.267	1.023	0.102
Hb (13,14]	0.289	0.488	0.231
Hb (14,15]	0.289	0.613	0.163
Hb (15,16]	0.175	0.403	0.122
Hb (16,∞)	0.131	0.382	0.306
Hb [11,15]	0.007	−0.120	0.050

${}^{*}$*p*< 0.1; ${}^{**}$ *p*< 0.05; ${}^{***}$ *p*< 0.01. [*] This table summarizes the association between Hb levels and the different dependent variables. Although not shown in the table, the rest of the explanatory variables were also included.
